# The SDN Approach for the Aggregation/Disaggregation of Sensor Data

**DOI:** 10.3390/s18072025

**Published:** 2018-06-25

**Authors:** Yi-Bing Lin, Shie-Yuan Wang, Ching-Chun Huang, Chia-Ming Wu

**Affiliations:** Department of Computer Science, National Chiao Tung University, Hsinchu 300, Taiwan; shieyuan@cs.nctu.edu.tw (S.-Y.W.); d3350233.cs05g@nctu.edu.tw (C.-C.H.); pcmwu.g@gmail.com (C.-M.W.)

**Keywords:** aggregation, disaggregation, Internet of Things, programmable switch, P4, sensor data, Software Defined Networking

## Abstract

In many Internet of Things (IoT) applications, large numbers of small sensor data are delivered in the network, which may cause heavy traffics. To reduce the number of messages delivered from the sensor devices to the IoT server, a promising approach is to aggregate several small IoT messages into a large packet before they are delivered through the network. When the packets arrive at the destination, they are disaggregated into the original IoT messages. In the existing solutions, packet aggregation/disaggregation is performed by software at the server, which results in long delays and low throughputs. To resolve the above issue, this paper utilizes the programmable Software Defined Networking (SDN) switch to program quick packet aggregation and disaggregation. Specifically, we consider the Programming Protocol-Independent Packet Processor (P4) technology. We design and develop novel P4 programs for aggregation and disaggregation in commercial P4 switches. Our study indicates that packet aggregation can be achieved in a P4 switch with its line rate (without extra packet processing cost). On the other hand, to disaggregate a packet that combines *N* IoT messages, the processing time is about the same as processing *N* individual IoT messages. Our implementation conducts IoT message aggregation at the highest bit rate (100 Gbps) that has not been found in the literature. We further propose to provide a small buffer in the P4 switch to significantly reduce the processing power for disaggregating a packet.

## 1. Introduction

Many Internet of Things (IoT) technologies have been used in applications for people flow, traffic flow, logistics flow, money flow, smart home, interactive art design, and so on. To promote IoT applications on campus, National Chiao Tung University (NCTU) is deploying several smart campus applications. These applications are created based on IoTtalk [1,2,3,4], an IoT application management platform that can be installed on top of IoT protocols such as AllJoyn [5], OM2M [6], OpenMTC [7], and an arbitrary proprietary protocol.

**Figure 1:**
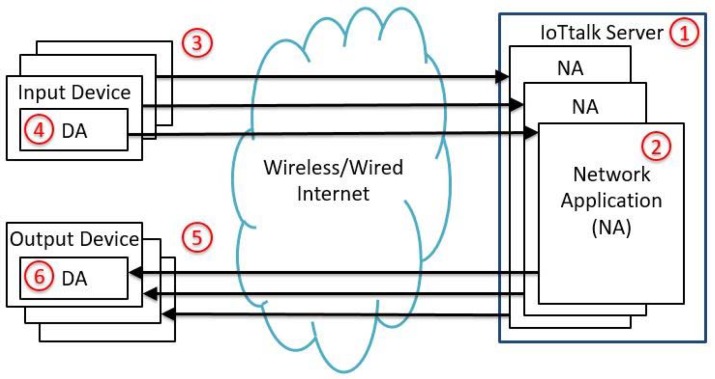
Simplified IoTtalk network architecture.

IoTtalk allows a designer to quickly establish connections and meaningful interactions between IoT devices. Figure 1 illustrates the simplified IoTtalk network architecture. In this client-server architecture, the IoT devices (the clients) are connected to the IoTtalk engine (the server; Figure 1(1)) in the Internet through wireline or wireless technologies. When an IoT device is connected to the IoTtalk server, the server automatically creates the network application (Figure 1(2)) for the device to interact with other devices. The IoT device can be a group of sensors (such as color sensor and temperature sensor) or controllers (such as switches and buttons), which is called an input device (Figure 1(3)). The device is installed as a device application (DA; Figure 1(4)) that collects the sensor data generated by the sensor/controller of the device. The DA then sends the sensor data to the IoTtalk server for processing (see path (4) → (2) in Figure 1). Similarly, an IoT device is called an output device (Figure 1(5)) if it is a group of actuators (e.g., robot arm). The DA of the output device receives data from the IoTtalk engine (see path (2) → (6) in Figure 1) to drive its actuators.

**Figure 2:**
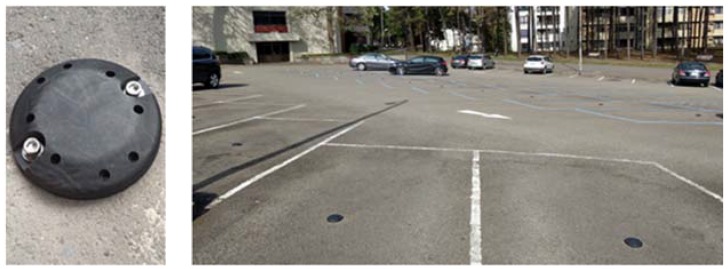
Smart parking in NCTU campus.

**Figure 3:**
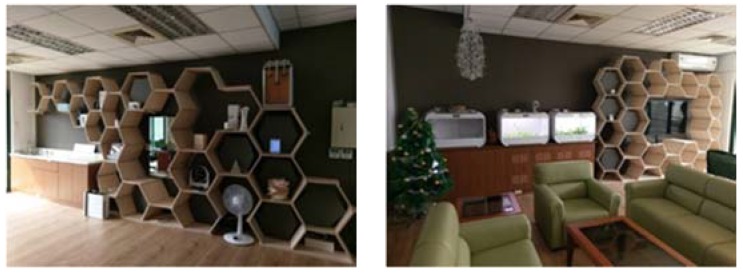
Room 311 in the MIRC building.

Based on IoTtalk, we have developed outdoor applications using LoRA-based PM2.5 detection [8] and NB-IoT-based parking (Figure 2), an emergency button, and dog tracking [9]. Besides outdoor applications, we have also created indoor IoT applications at the guest houses, the student dorms, and a research laboratory called Room 311 in the MIRC building (Figure 3). More than 100 sensors and actuators are installed in Room 311, and data are collected every day for machine-learning analysis. In addition, the smart home appliances are being installed in the newly built Graduate Dormitory 3. This student dorm will accommodate 1200 graduate students. A large amount of data (IoT messages) have been collected in Room 311, MIRC, and Graduate Dormitory 3. These IoT messages are UDP packets with small payloads. For example, inside an elevator car, we have installed a 3D accelerometer, a barometer, and a thermometer. Outside the car, the motor is attached to a biaxial vibration accelerator. The volume of data produced by these sensors is about 12 MB/minute. There are about 100 elevator cars on campus, which produce data at the speed of 20 MB/second. There are over 20 IoT applications exercised in NCTU, and these applications generate large volumes of small-packet data through path (4) → (2) in Figure 1.

To reduce the number of sensor data (IoT message) delivered from the IoT devices to the IoT server, a promising approach is to aggregate several small IoT messages into a large packet before they are delivered through the network [10]. When the packets arrive at the destination (in our example, the IoTtalk server is installed in the NCTU’s private cloud at the computer center), they are disaggregated into the original IoT messages. In this paper, we show how IoT data can be effectively aggregated and disaggregated. Specifically, we consider the software defined networking (SDN) approach to design IoT data aggregation and disaggregation for IoTtalk.

The paper is organized as follows. Section 2 introduces the programmable SDN switches. Section 3 describes how programmable SDN switches can be utilized for sensor data aggregation and disaggregation. Section 4 shows how to improve disaggregation performance for the SDN switches. Section 5 compares our solution with the previous studies. Specifically, we show an aggregation solution based on SDN controllers. Finally, Section 6 concludes our work and points out a future research direction.

## 2. Programmable SDN Switch

To resolve the sensor data delivery issue, Software Defined Networking (SDN) is considered as a promising solution. SDN decouples the data and the control planes [11]. Recently, programmability of the data plane has become one of the most desirable SDN features, which allows the user to describe how to process the packets in an SDN switch. This task can be achieved by using tools such as Programming Protocol-Independent Packet Processor (P4). P4 is a reconfigurable, multi-platform, protocol and target-independent packet processing language, which is used to facilitate dynamically programmable and extensible packet processing in the SDN data plane [12,13,14]. In SDN, a switch uses a set of “match+action” flow tables to apply rules for packet processing, and P4 provides an efficient way to configure the packet processing pipelines to achieve this goal. In a typical communications network, a packet consists of a packet header and the payload, and the header includes several fields defined by the network protocol. A P4 program describes how packet headers are parsed and their fields are processed by using the flow tables, in which the matched operations may modify the header fields of packets or the content of the metadata registers.

**Figure 4:**
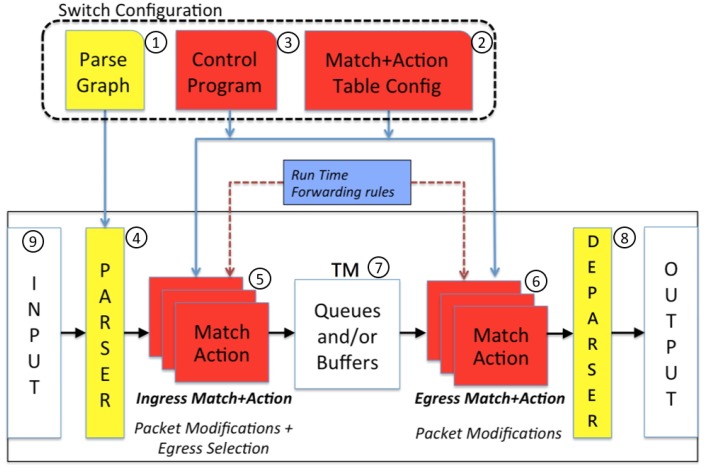
The P4 abstract forwarding model.

Figure 4 illustrates the P4 abstract forwarding model elaborated in [15] (the conference paper version of this paper), and the details are reiterated here for the reader’s benefit. In this model, a configuration file describes all components declared in the P4 program, including the parse graph (Figure 4(1)), the table configuration (Figure 4(2)), and an imperative control program that defines the order of the tables to be applied in the processing flow (Figure 4(3)). This configuration file is loaded into the switch to specify the parser (Figure 4(4)) followed by the flow tables in the ingress and the egress pipelines (Figure 4(5),(6)). The ingress pipeline generates an egress specification that determines the set of ports (and number of packet instances for each port) to which the packet will be sent. Between these two pipelines, there is a traffic manager (TM; see Figure 4(7)) that performs the queueing functions for the packets. At the TM, the packets are queued before being sent to the egress pipeline, through which the packet header may be further modified. At the deparser (Figure 4(8)), the headers are assembled back to a well-formed packet.

The parser extracts the header fields and the payload of an incoming packet following the parse graph defined in the P4 program. In P4, the parser state transitions are driven by the values of the fields in the headers.

Based on the above P4 forwarding model, we propose a novel approach that utilizes the header manipulation of the P4 switch to aggregate small packets into large packets and disaggregate large packets back into small packets. We describe our approach through the software switch behavior model bmv2, and actually implemented our algorithm in two EdgeCore P4 switches, which use barefoot’s Tofino P4 chips. The bmv2 framework enables the developers to implement P4 applications on a software switch. This framework is the de-facto architecture for most developers, as it is roughly equivalent to the abstract forwarding model illustrated in Figure 4. The Tofino chip is based on the Protocol Independent Switch Architecture (PISA) and can be programmed using P4. With the Capilano Software Development Environment (SDE) P4 compiler toolchain, the developers can compile and run P4 programs at a Tofino P4 switch at the line rate up to 100 Gbps per port. There are 64 ports in a P4 chip, which sum up to 6.5 Tbps.

## 3. Performance of IoT Packet Aggregation and Disaggregation in the P4 Switch

**Figure 5:**
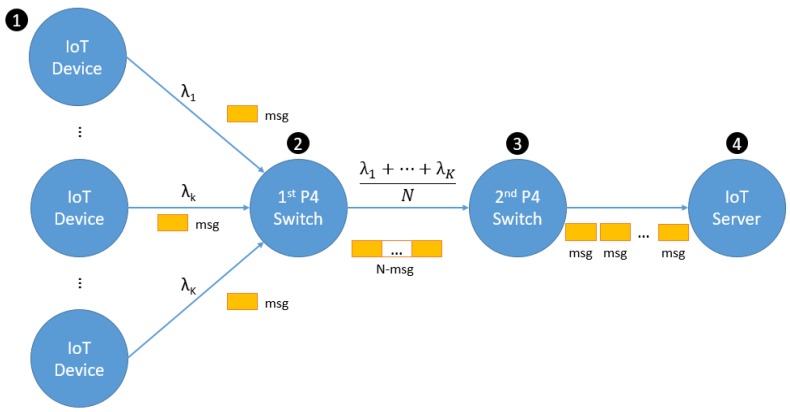
The P4 switch network architecture for IoT packet aggregation and disaggregation.

We have integrated IoTtalk in the SDN environment. The architecture will be illustrated in Section 5 later. In this section, Figure 5 illustrates a simplified general network architecture for IoT packet aggregation and disaggregation. In this architecture, *K* IoT devices (Figure 5(1)) send small packets (the IoT messages) to the IoT server (Figure 5(4)) though the P4 switches (Figure 5(2),(3)). When *N* consecutive IoT messages arrive at the first P4 switch, they are aggregated into a large packet. After aggregation, the first P4 switch sends the aggregated packet to the second P4 switch. The second P4 switch then disaggregates the packet into the original IoT messages, and sends them to the IoT server. Between these two P4 switches, there may be a wide area network not shown in the figure.

**Figure 6:**
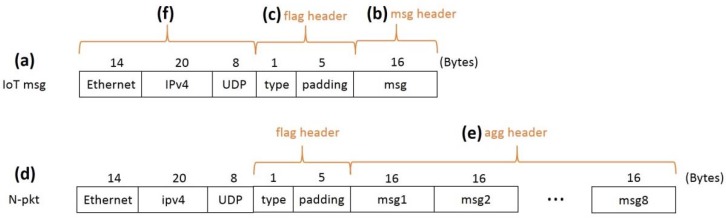
The packet formats of an IoT message and an *N*-packet (*N* = 8).

In our approach, the first P4 switch aggregates *N* IoT messages into one packet. Denote the aggregated packet as an *N*-packet. Figure 6 shows the packet formats of an IoT message and an *N*-packet for *N* = 8. For illustration purposes, we assume that an IoT message (Figure 6a) is a UDP packet with a 16-byte payload. In our design, the 16-byte payload is treated as a header called msg (Figure 6b). Between the UDP header and the payload is a 6-byte flag header (Figure 6c), of which the “type” field is used to indicate if the packet is an IoT message or an *N*-packet. The flag header is declared as

header_type flag_t {
fields {  
  type: 8;
     padding: 40;
}    
}         
header flag_t flag;

Note that the flag header has a 40-bit “padding” field to make the minimum length of an Ethernet frame 64 bytes. To meet this requirement, we purposely pad the length of the flag header to be 6 bytes so that together with a 14-byte Ethernet header, a 20-byte IP header, an 8-byte UDP header, and a 16-byte msg, the total length of an IoT message is 64 bytes. For an *N*-packet (Figure 6d), we consider the payloads aggregated from the *N* consecutive IoT messages as a header called agg (Figure 6e). That is, the *n*-th header field $msg_{n}$ is the payload of the *n*-th IoT message (in this example, 1 ≤ *n* ≤ *N* = 8). Therefore, the agg header is declared as

header_type agg_msg_t {
fields {   
    msg1: 128;
    msg2: 128;
    msg3: 128;
    msg4: 128;
    msg5: 128;
    msg6: 128;
    msg7: 128;
    msg8: 128;
}      
}          
header agg_msg_t agg;

We treat the payloads of a packet as a header and showed how to use the registers and the pipelines of the first P4 switch to conduct header operations to transform the payloads of *N* IoT messages (Figure 6b) into the payload of an aggregated packet (Figure 6e). When an IoT message arrives, the payload is parsed as a header. At Figure 4(4), the parser processes the Ethernet, the IP, and the UDP header (Figure 6f). When the flag_t header (Figure 6c) is encountered, the parser checks the value of the “type” field, then the parser extracts either the msg header (if “type” is 0xfa) or the agg header (if “type” is 0xfc). The P4 switch checks if the incoming packet is an IoT message (i.e., whether it has a valid msg header). If so, the payload of the message is considered as a header field that is saved in the register. The IoT message is then dropped. The P4 switch continues to collect the payloads of the incoming IoT messages in the register. When *N* payloads have been collected, these saved payloads are considered as header fields to produce the aggregated payload (Figure 6e). The aggregated packet is then sent out of the switch.

In Figure 5, for 1 ≤ *k* ≤ *K*, device *k* generates the packets following an arbitrary process with the rate $\lambda_{k}$. Then, the net arrivals of the packets to the first P4 switch form a Poisson process with the arrival rate $\lambda =\sum_{k=1}^{K}\lambda_{k}$. Note that the Poisson property of merged input streams is observed in the multiple sources of IoT traffic in Room 311, which is consistent with the superposition property of the Poisson process [16]. We first show that the inter-arrival times of the *N*-packets arriving at the second P4 switch follow an *N*-stage Erlang distribution with the mean N/λ:

$$f_{N}\left(t_{N}\right)=\frac{\lambda^{N}t_{N}{}^{N-1}e^{-\lambda t_{N}}}{\left(N-1\right)!}$$

and its Laplace transform is

(1) $$f_{N}^{*}\left(s\right)=\int_{t_{N}=0}^{\infty }f_{N}\left(t_{N}\right)e^{-st_{N}}dt_{N}={\left(\frac{\lambda }{s+\lambda }\right)}^{N}$$

**Figure 7:**
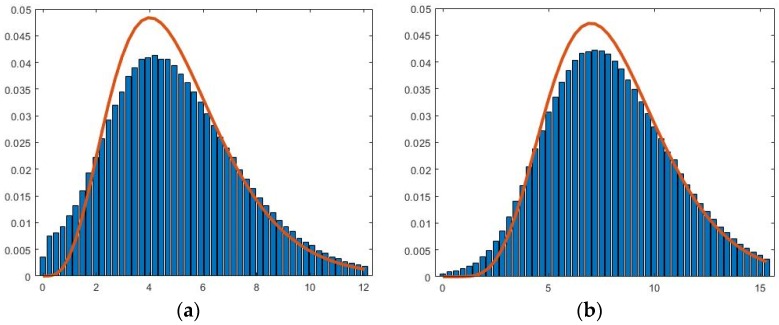

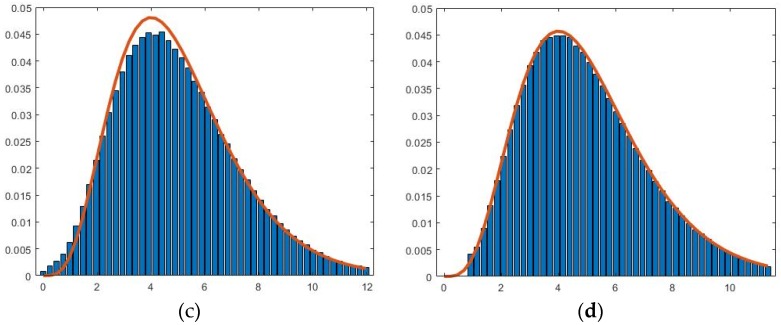
Validation of the Erlang departure process of the first P4 switch: (**a**) exponential (μ=1.1λ, *N* = 5); (**b**) exponential (μ=1.1λ, *N* = 8); (**c**) exponential (μ=2.2λ, *N* = 5); and (**d**) fixed (μ=1.1λ, *N* = 5).

Based on the Poisson property of merged input sources, we have conducted discrete event simulation to model the packet departures of the first P4 switch to see if the packet departures (i.e., the packet arrivals of the second P4 switch) follow the Erlang-*N* distribution. The first P4 switch is represented by either an M/M/1 or an M/G/1 queue in the simulation. Figure 7 compares the simulation results (the histogram bars) against the Erlang density function (the curves) in which the service times of the P4 switch are exponentially distributed (Figure 7a–c) or fixed (Figure 7d). In Figure 7a, the service rate μ=1.1λ and *N* = 5. The figure indicates that the departure process does fit Erlang-5 distribution. If we increase the service rate μ from 1.1λ to 2.2λ (Figure 7b) or increase the aggregation factor *N* from 5 to 8 (Figure 7c), the departure process fits the Erlang distribution better. Also, if the service times are fixed, then the departure fits the Erlang distribution better (Figure 7d). We note that for same kind of packets (e.g., the IoT messages in this paper), the service times are fixed in a P4 switch.

**Figure 8:**
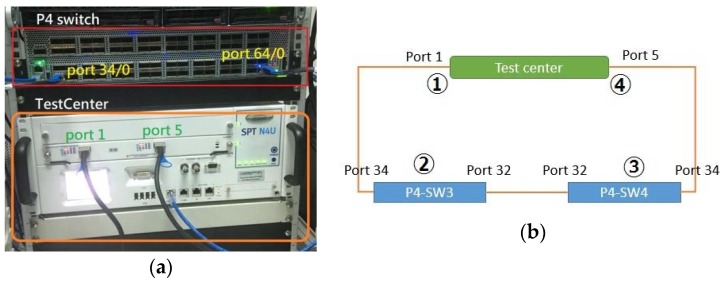
The experimental environment with 2 EdgeCore P4 switches and the TestCenter: (**a**) physical connection of the EdgeCore switch and the TestCenter and (**b**) functional block diagram.

The switches typically handle the packets without queueing, and its maximum processing capability per output port is given by its “line rate”. When the packets arrive within the line rate, no packet is dropped at the switch. We have actually implemented our algorithm in the EdgeCore P4 switches based on Barefoot Tofino chip (see Figure 8a).

Our study shows that for any *N* value, aggregation of the *N*-packets does not slow down the line rate of the first P4 switch (Figure 8b(2)). However, packet disaggregation at the second P4 switch (Figure 8b(3)) slows down packet processing of the switch. This phenomenon is due to the fact that the pipeline architecture is used, and to disaggregate an *N*-packet requires going through the ingress pipeline for *N* times.

On the other hand, the packet header overhead decreases as *N* increases. Specifically, for the example in Figure 6, the size of an *N*-packet is

(2) $$S_{N}=48+16N$$

in which 48 bytes come from the Ethernet (14 bytes), the IPv4 (20 bytes), the UDP (8 bytes), and the flag (6 bytes) headers, and every agg header contributes 16 bytes (Figure 6). From (2), the payload to packet ratio

(3) $$p_{N}=\frac{16N}{S_{N}}=\frac{N}{4+N}$$

In (3), $p_{N}$ is the ratio of the useful information delivered through the switch. If the throughput of a P4 switch is *X*, then we can define the goodput as

(4) $$X_{N}=p_{N}X\;\mathrm{for}\;N\geq 1$$

The network architecture in Figure 5 is actually implemented in Figure 8, in which two EdgeCore P4 switches are connected to serve as P4 switches 1 and 2 in Figure 8b(2),(3). The Spirent TestCenter [17] provides the traffic sources (Figure 8b(1)) to pump the combined IoT message traffic to the first EdgeCore P4 switch. Spirent TestCenter is an end-to-end testing solution that delivers high performance with deterministic answers. We use it to serve as the 100 Gbps traffic source, which is the highest speed in the world. The TestCenter also serves as the IoT server (Figure 8(4)), which receives the packets disaggregated at the second EdgeCore P4 switch. With the Spirent TestCenter, we are able to pump up to 100 Gbps line rate of packets in the experiments.

**Figure 9:**
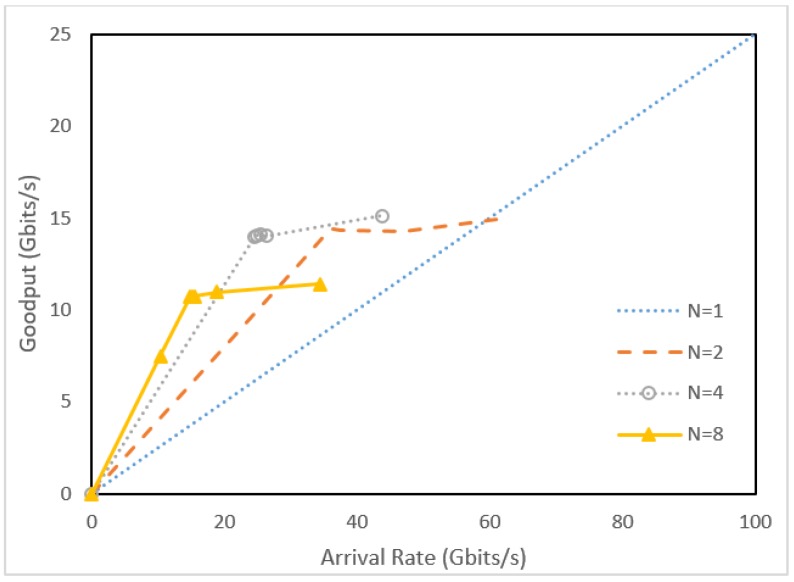
Goodput vs. packet arrival rates for the P4 switch configuration (1 ≤ *N* ≤ 8).

Based on the throughput measurements *X* of our experiments, Figure 9 shows the goodput $X_{N}$ against the net packet arrival rate λ from the IoT devices (Figure 5(1)) for different *N* values. The figure indicates that as *N* increases, the goodput increases. However, the $X_{N}$ saturates as λ increases. For a large *N*, $X_{N}$ saturates faster. This phenomenon is due to the hardware constraint of the P4 switch. The line rate for a port of the switch is 100 Gbps.

The service rate $\mu_{N}$ for disaggregating an *N*-packet cannot be directly measured from the P4 switch. Instead, by using the arrival rate and the goodput in Figure 9, we can use the G/D/1/1 queue to model the P4 switch to derive the disaggregation processing time for an *N*-packet. We use the goodput when there is no packet loss and without buffering as the service rate. Observing from the measurements of packet disaggregation of the P4 switch implementing our mechanism, we have

(5) $$\frac{1}{\mu_{N}}\approx \frac{\alpha_{N}}{\mu_{1}}$$

in which $0.85\leq \alpha_{N}\leq 1.14$. In other words, the processing time for disaggregating an *N*-packet is roughly the same as that for processing *N* IoT messages.

## 4. Packet Disaggregation in the P4 Switch with Queueing

This section shows how the queueing mechanism can effectively reduce the computing power of the P4 switch required to perform disaggregation of the IoT packets.

The previous section considers the case where the switch is operated without the input buffers, which is a typical design of switch. However, in our design for the disaggregation process, an *N*-packet needs to be looped back to the input port *N* times through the pipeline (Figure 4(5),(6)). Each time, an IoT message is extracted from the *N*-packet and sent out of the second P4 switch (Figure 4(8)). Due to this design, the resulting data rate to an input port is *N* times of the data rate of the *N*-packets. If the resulting data rate is greater than the line rate of the input port during some time intervals and there is no buffer in the input port, the looped *N*-packets will have a high chance of being dropped. In contrast, if some buffers can be provided in the input port, then packet drops during the disaggregation process can be reduced. With buffers provided at the input port, some queueing effects at the input port are observed as follows.

Based on the queueing theory, the second P4 switch can be designed to accommodate the traffic $\rho_{N}=\lambda /(N\mu_{N})$ if there is no queueing effect when the packets arrive at the rate λ/N. In the real world, the queueing effect cannot be avoided at the switch if the packets arrive at random. Therefore, we say that the second P4 switch can accommodate traffic $\rho_{N}$ if the expected waiting time $E[t_{W}]=\beta /\mu_{N}$ for a small β value (e.g., β=0.1).

Denote the service rate (the line rate) of the second P4 switch as $\mu_{1}$. If the packets are not aggregated, then the packet arrivals to the second P4 switch are a Poisson process with the rate λ. If the packets are aggregated into *N*-packets, then the packet arrival rate to the second switch is λ/N, and the packet processing rate $\mu_{N}$ is expressed in (5). With a small buffer time $\beta /\mu_{N}$ or the buffer size ⌈β⌉, we expected a smaller $\mu_{N}$ to keep the same goodput as that for $\mu_{1}$, and therefore the saved computing power can be allocated for other P4 traffics.

To validate the analytic and the simulation models, let the processing time of the second P4 switch for an *N*-packet have an exponential distribution with the rate $\mu_{N}$. After we have validated the simulation model, the exponential assumption is relaxed to accommodate fixed processing time that fits the real P4 switch operation. The $\mu_{N}$ value obtained in the previous section is used. The study in the previous section indicates that the inter-arrival times of the *N*-packets to the second P4 switch have the Erlang-*N* distribution with the rate λ/N. Then, the second P4 switch can be modeled as an $E_{N}/M/1$ queue by assuming that the packet processing time for an *N*-packet is exponentially distributed. This model is used to validate the simulation experiments. Then, we use the validated simulation to investigate the configuration in Figure 5 (i.e., Figure 8b) with the fixed processing times in the second EdgeCore P4 switch. Let $t_{R}$ and $t_{W}$ be the response and the waiting times of an *N*-packet at the second P4 switch, respectively. Then, from [16] we have

(6) $$E\left[t_{W}\right]=\frac{x}{\mu_{N}\left(1-x\right)}\;\mathrm{and}\;E\left[t_{R}\right]=E\left[t_{W}\right]+\frac{1}{\mu_{N}}=\frac{1}{\mu_{N}\left(1-x\right)}$$

From (1), *x* is expressed as

(7) $$x=f_{N}^{*}\left(\mu_{N}\left(1-x\right)\right)={\left[\frac{\lambda }{\mu_{N}\left(1-x\right)+\lambda }\right]}^{N}={\left(\frac{\theta }{1-x+\theta }\right)}^{N}$$

and $\theta =\lambda /\mu_{N}$. For *N* = 2, (7) is solved to yield

(8) $$x=\frac{1+2\theta -\sqrt{1+4\theta }}{2}=\frac{\mu_{2}+2\lambda -\sqrt{\mu_{2}\left(\mu_{2}+4\lambda \right)}}{2\mu_{2}}$$

Substitute (8) to (6) to yield

(9) $$E\left[t_{W}\right]=\frac{\frac{\mu_{2}+2\lambda -\sqrt{\mu_{2}\left(\mu_{2}+4\lambda \right)}}{2\mu_{2}}}{\mu_{2}\left[1-\frac{\mu_{2}+2\lambda -\sqrt{\mu_{2}\left(\mu_{2}+4\lambda \right)}}{2\mu_{2}}\right]}=\frac{\mu_{2}+2\lambda -\sqrt{\mu_{2}\left(\mu_{2}+4\lambda \right)}}{\mu_{2}\left[\mu_{2}-2\lambda +\sqrt{\mu_{2}\left(\mu_{2}+4\lambda \right)}\right]}$$

For *N* = 3, (7) is rewritten as

$$x^{4}-3\left(1+\theta \right)x^{3}+3{\left(1+\theta \right)}^{2}x^{2}-{\left(1+\theta \right)}^{3}x+\theta^{3}=0$$

By extracting the factor (*x* − 1) of the above equation, we have

$$\left(x-1\right)\left[x^{3}-\left(2+3\theta \right)x^{2}+\left(1+3\theta +3\theta^{2}\right)x-\theta^{3}\right]=0$$

which solves to yield

(10) $$\begin{array}{ll} x & =\frac{\left(2+3\theta \right)}{3}+\sqrt[{3}]{V+\sqrt[{2}]{V^{2}+W^{3}}}+\sqrt[{3}]{V-\sqrt[{2}]{V^{2}+W^{3}}}\\ & =\left(\frac{2}{3}+\frac{\lambda }{\mu_{3}}\right)+\sqrt[{3}]{V+\sqrt[{2}]{V^{2}+W^{3}}}+\sqrt[{3}]{V-\sqrt[{2}]{V^{2}+W^{3}}} \end{array}$$

in which

$$\begin{array}{ll} V & =\frac{-\left(2+3\theta \right)\left(1+3\theta +3\theta^{2}\right)}{6}+\frac{{\left(2+3\theta \right)}^{3}}{27}+\frac{\theta^{3}}{2}\\ & =\frac{-\left(2+\frac{9\lambda }{\mu_{3}}+27{\left(\frac{\lambda }{\mu_{3}}\right)}^{2}\right)}{54}\\ & =\frac{2\mu_{3}{}^{2}+9\lambda \mu_{3}+27\lambda^{2}}{54\mu_{3}{}^{2}} \end{array}$$

and

$$\begin{array}{ll} W & =\frac{\left(1+3\theta +3\theta^{2}\right)}{3}-\frac{{\left(2+3\theta \right)}^{2}}{9}\\ & =-\frac{3\theta +1}{9}\\ & =-\frac{3\lambda +\mu_{3}}{9\mu_{3}} \end{array}$$

Note that from the Galois’s theorem, (7) does not have a close form for *N* > 3 and must be computed numerically. As we pointed out previously, the second P4 switch can accommodate traffic $\rho_{N}$ if we provide a small buffer space β to satisfy the requirement $E[t_{W}]=\beta /\mu_{N}$. In other words, if the second P4 switch can accommodate $\rho_{N}$, its serving rate $\mu_{N}$ (computing power) must satisfy

(11) $$E\left[t_{W}\right]\leq \frac{\beta }{\mu_{N}}$$

Suppose that the original design of the second P4 switch targets at the line rate $\mu_{1}$, in which the packets are not aggregated, and every packet is processed at a fixed service time $1/\mu_{1}$. We also consider exponential service time with the same rate $1/\mu_{1}$ for the comparison and validation purpose. For an arbitrary packet arrival rate λ, if we want to keep the line rate $\mu_{1}$ to the switch without packet aggregation/disaggregation, then the inequality (11) must be satisfied, that is, from the response time of an M/M/1 queue, we have

(12) $$\frac{\lambda }{\mu_{1}\left(\mu_{1}-\lambda \right)}\leq \frac{\beta }{\mu_{1}}\;\mathrm{or}\;\mu_{1}\geq \left(\frac{\beta +1}{\beta }\right)\lambda$$

If the packet processing time is a fixed value, then from the response time of an M/D/1 queue, we have

(13) $$\frac{\lambda }{2\mu_{1}\left(\mu_{1}-\lambda \right)}\leq \frac{\beta }{\mu_{1}}\;\mathrm{or}\;\mu_{1}\geq \left(\frac{2\beta +1}{2\beta }\right)\lambda$$

Let $\mu_{1}^{*}(\beta )$ be the minimal $\mu_{1}$ that satisfies (12) and (13). Then we have

(14) $$\mu_{1}^{*}\left(\beta \right)=\{\begin{array}{ll} (\frac{\beta +1}{\beta })\lambda & \mathrm{for}\;\mathrm{Exponential}\;\mathrm{service}\;\mathrm{times}\\ \left(\frac{2\beta +1}{2\beta }\right)\lambda & \mathrm{for}\;\mathrm{fixed}\;\mathrm{service}\;\mathrm{times} \end{array}$$

Equation (14) indicates that the system with the fixed service times can process packets with less computing power than that for exponential service times. We have developed event-driven simulation to model the second P4 switch’s behavior. The event driven simulation is the same as those for the G/D/1 and the G/M/1 queues built in [18,19,20,21], and the details are omitted. Equation (14) is used to validate the simulation model. Our experiments indicate that the discrepancies between the analytic and the simulation results are within 0.4%.

Now, we consider the case in which the packets are aggregated into 2-packets at the first P4 switch, and are sent to the second P4 switch for disaggregation. That is, for *N* = 2, substitute (9) into (11) to yield

$$\frac{\mu_{2}+2\lambda -\sqrt{\mu_{2}\left(\mu_{2}+4\lambda \right)}}{\mu_{2}\left[\mu_{2}-2\lambda +\sqrt{\mu_{2}\left(\mu_{2}+4\lambda \right)}\right]}\leq \frac{\beta }{\mu_{2}}$$

which is simplified as

$$\left[\lambda -\frac{\beta \mu_{2}}{1+\beta }-\left(\sqrt{\beta^{2}+\beta }\right)\left(\frac{\mu_{2}}{1+\beta }\right)\right]\left[\lambda -\frac{\beta \mu_{2}}{1+\beta }+\left(\sqrt{\beta^{2}+\beta }\right)\left(\frac{\mu_{2}}{1+\beta }\right)\right]\leq 0$$

The above inequality implies

$$\lambda -\frac{\beta \mu_{2}}{1+\beta }-\left(\sqrt{\beta^{2}+\beta }\right)\left(\frac{\mu_{2}}{1+\beta }\right)\leq 0$$

which is solved to yield

$$\lambda \leq \left(\frac{\beta +\sqrt{\beta^{2}+\beta }}{1+\beta }\right)\mu_{2}$$

Let $\mu_{2}^{*}(\beta )$ be the minimal $\mu_{2}$ that satisfies the above inequality. Then,

(15) $$\mu_{2}^{*}\left(\beta \right)=\frac{\left(1+\beta \right)\lambda }{\beta +\sqrt{\beta^{2}+\beta }}$$

Similarly, for *N* = 3, substitute (10) into (11) to yield

$$\frac{\left(2\mu_{3}+3\lambda \right)+3\mu_{3}\left(\sqrt[{3}]{V+\sqrt[{2}]{V^{2}+W^{3}}}+\sqrt[{3}]{V-\sqrt[{2}]{V^{2}+W^{3}}}\right)}{\mu_{3}\left[\mu_{3}-3\lambda -3\mu_{3}\left(\sqrt[{3}]{V+\sqrt[{2}]{V^{2}+W^{3}}}+\sqrt[{3}]{V-\sqrt[{2}]{V^{2}+W^{3}}}\right)\right]}\leq \frac{\beta }{\mu_{3}}$$

Let $\mu_{3}^{*}(\beta )$ be the minimal $\mu_{3}$ that satisfies the above inequality. Then,

(16) $$\mu_{3}^{*}\left(\beta \right)=\frac{3\left(1+\beta \right)\lambda }{\left(\beta -2\right)-3\left(1+\beta \right)\left(\sqrt[{3}]{V+\sqrt[{2}]{V^{2}+W^{3}}}+\sqrt[{3}]{V-\sqrt[{2}]{V^{2}+W^{3}}}\right)}$$

Equations (15) and (16) are used to validate the simulation model again. Our experiments indicate that the discrepancies between the analytic and the simulation results are within 0.9%.

**Figure 10:**
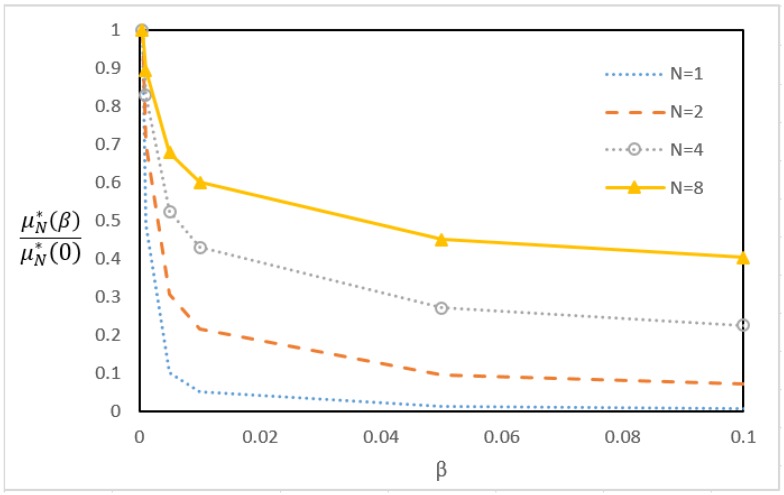
$\mu_{N}^{*}\left(\beta \right)$ against *N* and β.

After validation by (15) and (16), the simulation model, together with the measured $\mu_{N}^{*}(\beta )$ obtained from Figure 9 and Equation (5), are used to compute $\mu_{N}^{*}(\beta )$, the minimal $\mu_{N}$ that satisfies (11). Figure 10 plots $\mu_{N}^{*}(\beta )$ against the aggregation factor *N* and the queueing factor β, in which $\mu_{N}^{*}(\beta )$ is normalized by $\mu_{N}^{*}(0)$. The figure indicates that a queueing mechanism with a small buffer can significantly reduce the computation power required to handle disaggregation of the IoT packets. Specifically, when β≤0.02, increasing β will significantly reduce $\mu_{N}^{*}(\beta )$. On the other hand, for β≥0.05, increasing the buffer size does not improve the performance. Therefore, a buffer of size ⌈β⌉=1 is good enough to reduce the processing rate to $\mu_{N}^{*}(\beta )$, which is not difficult to implement in the P4 chip (i.e., to create a buffer at Figure 4(9)).

## 5. Related Work and Aggregation/Disaggregation Based on SDN Controllers

In the existing solutions, packet aggregation/disaggregation is performed by software run at the CPUs of the servers. As we show in this section, the software solutions based on the CPU-based architecture will result in degraded goodput (and therefore degraded throughput). Many studies [10,22,23] described the benefits of packet aggregation in saving transmission overhead in the networks. However, none of them investigate the processing cost of a network node for aggregation/disaggregation. This section shows the aggregation/disaggregation cost in the servers based on the CPU architecture.

**Figure 11:**
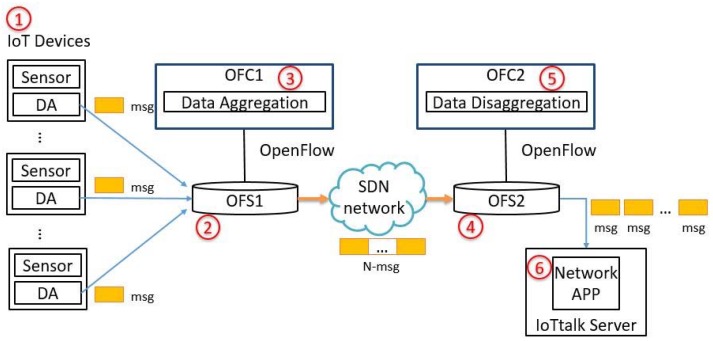
Sensor data aggregation/disaggregation for IoTtalk based on the SDN controllers (the CPU-based architecture).

The original design of SDN separates the control plane and the data plane of legacy switches so that the network nodes (e.g., SDN switches) are only responsible for data forwarding, which makes the network management simpler and more convenient. The SDN controller called the OpenFlow Controller (OFC) utilizes OpenFlow [24] to interact with the SDN switches called the OpenFlow Switches (OFSs). SDN network management introduces a flow control application that maintains the routing rules of the OFSs through the OFC. In this way, SDN makes the network more programmable, flexible, and dynamically manageable. Specifically, the SDN can dynamically adjust the data routing path to avoid network congestion. Figure 11 shows an aggregation/disaggregation mechanism for IoTtalk based on the SDN architecture, which is similar to the one illustrated in Figure 5, except that the P4 switches are replaced by the OpenFlow switches (which cannot be programmed to perform packet aggregation/disaggregation), and the OpenFlow Controllers are involved. In this architecture, *K* IoT devices (Figure 11(1)) are installed in Building 311 of the MIRC Building. The DAs of the devices collect the sensor data and send small packets (the IoT messages) to an OpenFlow Switch (OFS1; Figure 11(2)). OFS1 connects to an OpenFlow Controller (OFC1; Figure 11(3)) that is responsible for packet aggregation. OFC1 sends the aggregated packets to OFS1. These aggregated packets are sent to another OpenFlow Switch (OFS2; Figure 11(4)) through one or more SDN networks. OFS2 sends the packets to its controller (OFC2; Figure 11(5)) for packet disaggregation and outputs them to the IoTtalk server (Figure 11(6)). Both OFC1 and OFC2 are servers responsible for processing the packets.

**Figure 12:**
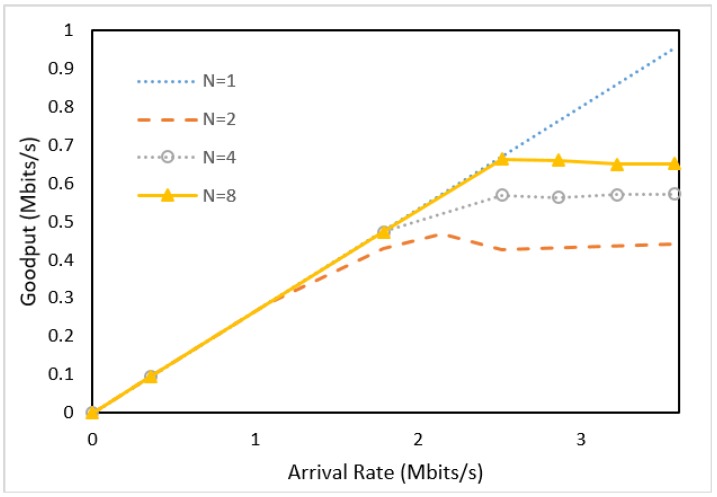
Goodput vs. packet arrival rates for the SDN controller configuration (1 ≤ *N* ≤ 8).

Figure 12 shows the goodput of aggregation/disaggregation processed by Figure 11(3),(5). The goodput curves indicate that in the CPU-based architecture (i.e., the OFC servers), aggregation/disaggregation processing overheads are significantly higher than those of the pipeline P4 architecture shown (Figure 9). Specifically, the goodputs for *N* ≥ 2 are worse than those for *N* = 1 in Figure 12. On the other hand, the goodputs for *N* ≥ 2 are much better than those for *N* = 1 in Figure 10.

## 6. Concluding Remarks

This paper proposed to utilize the P4 switch for quick packet aggregation and disaggregation. We designed and developed novel P4 programs for aggregation and disaggregation, which are implemented in commercial P4 switches. Our study indicated that packet aggregation can be achieved in a P4 switch with its 100 Gbps line rate (without extra packet processing cost). On the other hand, to disaggregate a packet that combines *N* IoT messages, the processing time is about the same as processing *N* individual IoT messages. We further proposed to provide a small buffer at the input ports of the P4 switch to significantly reduce the packet drop probability when disaggregating an *N*-packet. Also note that our implementation conducted IoT message aggregation at the highest bit rate (100 Gbps) that has not been found in the literature. As for the future work, we are considering new techniques to improve the performance of packet disaggregation. One possible technique is the multicast mechanism supported by the EdgeCore switch.

As a final remark, if the aggregation and the disaggregation P4 switches are indirectly connected through a network with bandwidth lower than their line rates, due to network congestion, when packets travel from the first P4 switch to the second P4 switch, some packets may be dropped while the aggregated *N*-packets are safely delivered to the second switch. In this case, together with the disaggregation process performed at the second P4 switch, the aggregation process performed at the first P4 switch effectively reduces the probability of IoT messages lost over the lower-bandwidth network.
